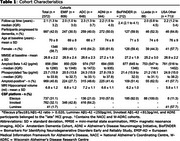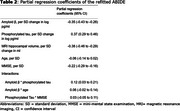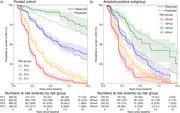# ABIDEing with automated cerebrospinal fluid assays; update of an MCI to dementia prediction model

**DOI:** 10.1002/alz70861_108352

**Published:** 2025-12-23

**Authors:** Pieter J. van der Veere, Argonde C. van Harten, Ingrid S. van Maurik, Charlotte E. Teunissen, Frederik Barkhof, Stephanie J. B. Vos, Lutz Frölich, Johannes Kornhuber, Jens Wiltfang, Wolfgang Maier, O. Peters, Eckart Rüther, Giovanni B. Frisoni, Luiza Spiru, Yvonne Freund‐Levi, Åsa K. Wallin, Harald Hampel, Magda Tsolaki, Iwona Kłoszewska, Patrizia Mecocci, Bruno Vellas, Simon Lovestone, Samantha Galluzzi, Sanna‐Kaisa Herukka, Isabel Santana, Inês Baldeiras, Alexandre Mendonca, Dina Silva, Gael Chételat, Géraldine Poisnel, Pieter Jelle Visser, Sterling C Johnson, Erik Stomrud, Oskar Hansson, Sebastian Palmqvist, Gerard Piñol‐Ripoll, Johannes Berkhof, Wiesje M. van der Flier

**Affiliations:** ^1^ Amsterdam Neuroscience, Neurodegeneration, Amsterdam Netherlands; ^2^ Alzheimer Center Amsterdam, Neurology, Vrije Universiteit Amsterdam, Amsterdam UMC location VUmc, Amsterdam Netherlands; ^3^ Department of Epidemiology and Data Science, Amsterdam UMC, Amsterdam Netherlands; ^4^ Amsterdam Neuroscience, Neurodegeneration, Amsterdam, Noord‐Holland Netherlands; ^5^ Neurochemistry Laboratory, Department of Clinical Chemistry, Amsterdam UMC, location VUmc, Amsterdam Netherlands; ^6^ Alzheimer Center Amsterdam, Department of Neurology, Amsterdam UMC, location VUmc, Amsterdam Netherlands; ^7^ Alzheimer Center Amsterdam, Department of Neurology, Amsterdam Neuroscience, Vrije Universiteit Amsterdam, Amsterdam UMC, Amsterdam Netherlands; ^8^ Amsterdam Public Health, Amsterdam, Noord Holland Netherlands; ^9^ Northwest Academy, Alkmaar, Noord holland Netherlands; ^10^ Neurochemistry Laboratory, Amsterdam Neuroscience, Program Neurodegeneration, Amsterdam UMC, Vrije Universiteit Amsterdam, Amsterdam, Noord‐Holland Netherlands; ^11^ University College London, London UK; ^12^ Amsterdam Neuroscience, Brain Imaging, Amsterdam Netherlands; ^13^ Department of Radiology and Nuclear Medicine, Vrije Universiteit Amsterdam, Amsterdam University Medical Center, location VUmc, Amsterdam Netherlands; ^14^ Alzheimer Center Limburg, School for Mental Health and Neuroscience, Maastricht University, Maastricht, Limburg Netherlands; ^15^ Central Institute of Mental Health, Medical Faculty Mannheim, University of Heidelberg, Mannheim Germany; ^16^ Department of Psychiatry and Psychotherapy, Universitätsklinikum Erlangen and Friedrich‐Alexander Universität Erlangen‐Nürnberg, Erlangen Germany; ^17^ German Center for Neurodegenerative Diseases (DZNE), Göttingen Germany; ^18^ Department of Psychiatry and Psychotherapy, University Medical Center Goettingen, University of Goettingen, Goettingen Germany; ^19^ Friedrich‐Alexander University of Erlangen‐Nürnberg, Erlangen Germany; ^20^ Department of Neurodegenerative Diseases and Geriatric Psychiatry, University Hospital, Bonn Germany; ^21^ Department of Psychiatry, Charité—Universitätsmedizin Berlin, corporate member of Freie Universität Berlin, Berlin Germany; ^22^ German Center for Neurodegenerative Diseases, Berlin Germany; ^23^ Department of Psychiatry and Psychotherapy, University of Göttingen, Göttingen Germany; ^24^ Memory Clinic, Geneva University Hospitals, Geneva Switzerland; ^25^ Memory Clinic, Brain/Mental Health and Longevity Medicine, Ana Aslan International Foundation, Bucharest Romania; ^26^ Geriatrics, Gerontology Old Age Psychiatry and Longevity Medicine ‐ Clinical Department, Carol Davila University of Medicine and Pharmacy, Saint Luke’s Clinical Hospital, Bucharest Romania; ^27^ Karolinska Institutet, and Department of Geriatric Medicine, Karolinska University, Stockholm Sweden; ^28^ King's College London, London UK; ^29^ Örebro University, Örebro Sweden; ^30^ Lund University, Lund Sweden; ^31^ Sorbonne Université, AP‐HP, GRC n° 21, Alzheimer Precision Medicine (APM), Hôpital de la Pitié‐Salpêtrière, Paris France; ^32^ Aristotle University of Thessaloniki, Thessaloniki Greece; ^33^ Medical University of Lodz, Lodz Poland; ^34^ Division of Clinical Geriatrics, NVS Department, Karolinska Institutet, Stockholm Sweden; ^35^ Institute of Gerontology and Geriatrics, Department of Medicine and Surgery, University of Perugia, Perugia Italy; ^36^ Institute of Aging, Toulouse University Hospital ‐ UMR 1295, Toulouse France; ^37^ Department of Psychiatry, University of Oxford, Oxford UK; ^38^ IRCCS Istituto Centro San Giovanni di Dio Fatebenefratelli, Brescia Italy; ^39^ Institute of Clinical Medicine, University of Eastern Finland, Kuopio Finland; ^40^ Center for Neuroscience and Cell Biology, Faculty of Medicine, University of Coimbra, Coimbra, Coimbra Portugal; ^41^ Neurology Department, Centro Hospitalar e Universitário de Coimbra, Coimbra, ‐ Portugal; ^42^ Faculty of Medicine, University of Coimbra, Coimbra Portugal; ^43^ Department of Neurology, Centro Hospitalar e Universitário de Coimbra, Coimbra Portugal; ^44^ Faculty of Medicine, University of Lisbon, Lisbon, Lisbon Portugal; ^45^ Faculty of Medicine, University of Lisbon, Lisbon Portugal; ^46^ Institute of Molecular Medicine, University of Lisbon, Lisbon Portugal; ^47^ Centre for Biomedical Research, Universidade do Algarve, Faro Portugal; ^48^ Normandie Université, Université de Caen, Institut National de la Santé et de la Recherche Médicale, Caen France; ^49^ Normandie Univ, UNICAEN, INSERM, U1237, PhIND "Physiopathology and Imaging of Neurological Disorders", NeuroPresage Team, GIP Cyceron, Caen France; ^50^ Alzheimer Centrum Limburg, Maastricht University, Maastricht Netherlands; ^51^ Department of Medicine, University of Wisconsin‐Madison School of Medicine and Public Health, Madison, WI USA; ^52^ Wisconsin Alzheimer's Disease Research Center, Madison, WI USA; ^53^ Lund University, Malmö Sweden; ^54^ Memory Clinic, Skåne University Hospital, Malmö, Skåne Sweden; ^55^ Clinical Memory Research Unit, Lund University, Malmö, Skåne Sweden; ^56^ Skåne University Hospital, Malmö, 21428 Skåne Sweden; ^57^ Clinical Memory Research Unit, Department of Clinical Sciences Malmö, Faculty of Medicine, Lund University, Lund Sweden; ^58^ Unitat Trastorns Cognitius, Clinical Neuroscience Research, Santa Maria University Hospital, IRBLleida, Lleida Spain; ^59^ Universitari Santa Maria, Lleida Spain; ^60^ Department of Epidemiology and Biostatistics, Amsterdam university medical center, Amsterdam Netherlands; ^61^ Amsterdam Neuroscience, Vrije Universiteit Amsterdam, Amsterdam UMC, Amsterdam Netherlands

## Abstract

**Background:**

Automated cerebrospinal fluid (CSF) biomarker assays have largely replaced manual immunoassays for measuring amyloid pathology. Their relevance is increasing as amyloid‐targeting therapies (ATTs) are becoming available for amyloid‐positive mild cognitively impaired (MCI) individuals. Therefore, we refitted and validated the ABIDE model, predicting progression from MCI to dementia, with CSF measurements from the automated Elecsys platform. Additionally, we evaluated the performance in an amyloid‐positive subpopulation, potentially eligible for ATTs.

**Method:**

We combined data from MCI participants of seven single‐centre and multicentre observational cohorts: Amsterdam Dementia Cohort (*n* =648), Alzheimer's Disease Neuroimaging Initiative (*n* =544), BioFINDER (*n* =212), European Medical Information Framework for Alzheimer's Disease (*n* =809), Lleida (*n* =88), National Alzheimer's Coordinating Centre (*n* =63), and Wisconsin Alzheimer's Disease Cohort (*n* =9). Participants were included with MCI at baseline, a baseline Mini‐Mental State Examination, either a magnetic resonance imaging hippocampal volume or CSF Aβ1‐42 and pTau181 measurements, and at least six months of follow‐up. Elecsys was used in 737 (31%) participants. A Cox model was used to predict time to dementia using the variables in the previous ABIDE model (Maurik et al. 2019). Model discrimination and calibration were evaluated with leave‐one‐cohort‐out cross‐validation. Calibration was assessed in the pooled cohort (PC) and amyloid‐positive (APos) subgroup, stratified by predicted risk: PC/APos1 (<P16), PC/APos2 (P16‐P50), PC/APos3 (P50‐P86), PC/APos4 (>P86).

**Result:**

Of 2372 MCI participants (Table 1; 70±8yrs, 57%F; 41% amyloid‐positive) with a median follow‐up of 2.1yrs, 997 (42%; 563 [58%] amyloid‐positive) developed dementia (IQR:1.3‐3.2yrs). The refitted coefficients resemble the prior model, except for a larger effect of the Aβ1‐42*pTau interaction (Table 2). Discrimination was similar to the prior ABIDE model, with Harrell's C of 0.70 (95%CI:0.69‐0.71), and calibration was good in the pooled cohort, amyloid‐positive subgroup (Figure 1), and across CSF assays. In the amyloid‐positive subgroup, all four risk groups had a substantial progression risk with a median predicted progression time of 6.3yrs (95%CI:6.1‐6.6) in APos1, 3.7yrs (95%CI:3.5‐4.0) in APos2, 3.0yrs (95%CI:2.8‐3.0) in APos3, and 2.0yrs (95%CI:2.0‐2.1) in APos4.

**Conclusion:**

We updated the ABIDE model for predicting MCI to dementia progression with automated CSF measurements. The model was well calibrated in amyloid‐positive patients and may support clinical discussions regarding the initiation of ATTs.